# Successful Dissemination to Additional Populations Through Adaptation of the HAP-B Curriculum

**DOI:** 10.1093/geroni/igaf122.1115

**Published:** 2025-12-31

**Authors:** Sarah Punshon

**Affiliations:** William S. Middleton Memorial Veterans’ Hospital, Madison, Wisconsin, United States

## Abstract

Healthy Aging Project-Brain, Occupational Therapy (HAP-B OT) is an adaptation of the original HAP-B group designed to promote health lifestyle behaviors for older persons in subacute rehab, residential and/or other inpatient settings. HAP-B OT includes four standalone sessions that can be tailored to participants with a wide range of physical and cognitive abilities. We will describe the process of adapting HAP-B to create HAP-B OT using the 5-stage group model originally developed by Mildred Ross, OTR. This model was chosen because activities are sequenced intentionally to elicit an organized behavioral response that facilitates learning. Activities for this specific group were selected because they can be tailored in the moment to varying levels of cognitive and physical demands based on the abilities of each unique set of group members. This style of group is more inclusive of the varied physical and cognitive abilities of older adults typically treated in subacute rehab, residential, and/or other inpatient settings. HAP-B OT includes the same 4 healthy aging brain topics as the original HAP-B which are sleep, physical activity, social connection, and cognitive stimulation, and provides opportunities to practice skills learned during group sessions through engagement in meaningful daily living activities and after group sessions through SMART goal setting. Outcomes measured are focused on improving perceived self-efficacy, and include the Perceived Health Competence Scale-2 and the Perceived Stress Scale-4.

